# Intermittent viraemia and immune reconstitution in patients with more than 10–15 years of antiretroviral therapy: baseline values still matter

**DOI:** 10.7448/IAS.17.4.19689

**Published:** 2014-11-02

**Authors:** Gesa Erdbeer, Michael Sabranski, Ina Sonntag, Albrecht Stoehr, Heinz-A Horst, Andreas Plettenberg, Knud Schewe, Stefan Unger, Hans-J Stellbrink, Stefan Fenske, Christian Hoffmann

**Affiliations:** 1 University of Schleswig-Holstein, Campus Kiel, Kiel, Germany; 2 Infektionsmedizinisches Centrum Hamburg, Study Center, Hamburg, Germany; 3 IFI, Institute for Interdisciplinary Medicine, Hamburg, Germany

## Abstract

**Introduction:**

Data on patients with long-term exposure to ART is scarce because controlled studies usually do not follow up patients for more than five to seven years. We were interested whether baseline parameters such as CD4 T-cell nadir or pre-treatment viraemia do have an impact on ART success after more than a decade of treatment.

**Methods:**

ELBE is a cross-sectional study on adult HIV+ patients presenting consecutively with viral suppression (<50 HIV RNA copies/mL) and with ART exposure of at least five years. In this sub-analysis, all patients with more than 10 years of ART exposure were evaluated for immune reconstitution and for intermittent transient viraemia (50–1000 copies/mL, defined as “blips”) during the last five years.

**Results:**

From a total of 894 patients included in the three participating ELBE centres, 524 patients had an ART exposure of at least 10 years and had been treated continuously during the last 5 years. Of these, 33.4% had at least one “blip” while 63.5% did not show any transient viraemia of more than 50 copies/mL. Patients with at least one blip had a higher pre-treatment viraemia compared to patients without blips (5.30 versus 5.06 log copies/mL, p=0.0003). In patients with a pre-treatment viraemia of more than 100,000, 50,000–100,000 and less than 50,000 copies/mL, the proportions of patients with blips during the last five years were 39.5%, 30.5% and 21.8% (p=0.007), respectively. The history of an AIDS-defining illness or the CD4 T-cell nadir was not associated with a higher frequency of blips. However, CD4 T-cell nadir was a strong predictor for current CD4 T-cell counts. In patient groups with a CD4 T-cell nadir of 0–99, 100–199, 200–349, 350+ cells/µL, the median current CD4 T cells were 571, 667, 710 and 890 cells/µL, respectively. These differences remained significant when the analysis was restricted to patients with more than 15 years of ART exposure (n=268).

**Conclusions:**

In this large group of positively selected HIV+ patients with long-term exposure to ART of at least 10–15 years, high pre-treatment viraemia was still associated with a higher frequency of intermittent transient viraemia (“blip”). A low CD4 T-cell nadir remained associated with a lower CD4 cell recovery. The clinical implications of these findings remain to be evaluated.

